# IgG4-related disease presenting with hypercalcemia: case report and mechanistic insights

**DOI:** 10.3389/fimmu.2025.1720791

**Published:** 2025-12-10

**Authors:** Yaxin Li, Xing Niu, Ling Li, Zhangxue Hu

**Affiliations:** 1 Department of Nephrology, Institute of Kidney Diseases, West China Hospital, Sichuan University, Chengdu, Sichuan, China; 2 Department of Gastroenterology, West China Hospital, Sichuan University, Chengdu, Sichuan, China; 3 Department of Nephrology, Bozhou District People’s Hospital of Zunyi City, Zunyi, Guizhou, China

**Keywords:** IgG4-related disease (IgG4-RD), hypercalcemia, acute kidney injury, M2 macrophages, 1α-hydroxylase (CYP27B1)

## Abstract

Immunoglobulin G4-related disease (IgG4-RD) is a rare, multisystem disorder with diagnostic challenges. We report a case of IgG4-RD initially presenting with acute kidney injury (AKI) and hypercalcemia in a 61-year-old woman with a history of recurrent eyelid edema. On admission, serum creatinine and calcium were significantly elevated (414 μmol/L and 3.69 mmol/L, respectively). After excluding other etiologies, diagnosis was confirmed by renal biopsy and elevated serum IgG4 levels. Treatment with prednisone acetate and supportive care resulted in gradual clinical and laboratory improvement. This case underscores the importance of considering IgG4-RD in patients with unexplained AKI and hypercalcemia, highlighting the value of early diagnosis and targeted therapy. Furthermore, we explored potential pathophysiological mechanisms underlying hypercalcemia in IgG4-RD and found that M2 macrophages may overexpress 1α-hydroxylase, enhancing the conversion to active 1,25(OH)_2_D, and thereby increasing intestinal calcium absorption and leading to hypercalcemia, which has also been described in granulomatous diseases.

## Introduction

Immunoglobulin G4-related disease (IgG4-RD) is a chronic immune-mediated fibroinflammatory disorder characterized by multisystem involvement, including the pancreas, salivary glands, lacrimal glands, biliary tract, kidneys, retroperitoneum, lymph nodes, and large vessels (1, 2). Renal involvement occurs in approximately 10%– 15% of cases, predominantly as IgG4-related tubulointerstitial nephritis (IgG4-TIN), with renal insufficiency—acute or chronic—often being the initial presentation (3). Despite growing recognition in recent years, IgG4-RD remains challenging to diagnose due to its diverse clinical manifestations and overlap with malignancies, autoimmune diseases, and infectious conditions. Severe hypercalcemia is a rare manifestation and may lead to misdiagnosis or delayed treatment. Here, we report a rare case of IgG4-RD presenting with marked hypercalcemia and acute kidney injury (AKI). Diagnosis was established through comprehensive clinical, serological, and histopathological assessments. Mechanistic exploration suggested that the hypercalcemia in this case may have been associated with macrophage overactivation (4, 5).

## Case presentation

A 61-year-old woman with no significant past medical history presented in November 2024 with progressive bilateral eyelid and facial swelling, accompanied by exertional dyspnea and persistent productive cough with white sputum. Initial evaluation at a local hospital revealed elevated serum creatinine (sCr, 113 μmol/L). Despite empirical treatment with antibiotics and diuretics, her symptoms improved only partially. Over the following two months, facial edema recurred, accompanied by persistent renal dysfunction (sCr 238.7 µmol/L) and refractory hypercalcemia (serum calcium 4.19 mmol/L). On February 28, 2025, she was admitted to our hospital with worsening edema. Her medical history was notable for a 3-year history of hypertension, intermittently treated with valsartan, with no relevant family history.

Her vital signs on admission were as follows: height 151 cm, weight 52 kg (BMI 22.8 kg/m²), temperature 36.5 °C, pulse 91 beats/min, blood pressure 130/85 mmHg, and respiratory rate 20 breaths/min. Physical examination showed bilateral eyelid and facial edema without rash or lymphadenopathy, along with mild lower limb edema. Lung auscultation revealed slightly coarse breath sounds, while the remainder of the systemic examination was unremarkable.

Initial laboratory studies showed normal blood counts. Serum creatinine had increased to 414 μmol/L, with blood urea nitrogen (BUN) elevated at 29.3 mmol/L. Serum calcium, corrected for albumin, remained elevated at 3.69 mmol/L (normal range [NR], 2.11–2.52 mmol/L), while serum phosphorus and alkaline phosphatase levels were within normal limits. Parathyroid hormone (PTH) was also normal at 2.96 pmol/L. Urinalysis demonstrated 1+ proteinuria without hematuria, and the urine protein-to- creatinine ratio (UPCR) was 0.50 g/g Cr. Chest CT showed bilateral patchy pulmonary infiltrates with interstitial changes, multiple enlarged mediastinal and bilateral hilar lymph nodes (**Supplementary Figure S1**), and mild bilateral pleural effusions. Renal ultrasound demonstrated bilaterally small kidneys (right kidney, approximately 8.8×4.9×3.8cm; left kidney, approximately 8.5×4.9×4.7cm) with slightly increased echogenicity and mildly blurred corticomedullary differentiation.

Autoimmune screening was negative for antinuclear antibody (ANA), extractable nuclear antigens, anti-dsDNA antibodies, and antineutrophil cytoplasmic antibodies (ANCA), as assessed by both indirect immunofluorescence and chemiluminescence immunoassay. Complement components C3 and C4 were within the normal range. Given the bilateral hilar lymphadenopathy and hypercalcemia, serum angiotensin-converting enzyme (ACE) was also within the normal range (35.5 U/L; NR, 22–62 U/L), and both the tuberculosis interferon-γ release assay (TB-IGRA) and sputum cultures for Mycobacterium tuberculosis were negative. Serum immunoglobulin G (IgG) level was 10.94 g/L (NR, 8.60–17.40 g/L). Serum free κ/λ light chain ratio, serum protein electrophoresis (sPEP), and serum immunofixation electrophoresis (sIFE) showed no evidence of monoclonal proteins. Bone marrow biopsy revealed no abnormalities. Serum vascular endothelial growth factor (VEGF) was 58.51 pg/mL (NR, 0–142 pg/mL). Thyroid function tests were within normal limits. Serum immunoglobulin G4 (IgG4) was markedly elevated at 11.151 g/L (NR, 0.030–2.010 g/L), accompanied by significantly increased rheumatoid factor (RF, 725.0 IU/mL; NR <20 IU/mL), C-reactive protein (CRP, 10.90 mg/L; NR 0–6.0 mg/L), and erythrocyte sedimentation rate (ESR, 66.0 mm/h; NR <38 mm/h). In addition, a direct Coombs test was positive (2+). Hemoglobin was 136 g/L (NR, 115–150 g/L) on admission. Further hemolysis evaluation revealed normal haptoglobin and lactate dehydrogenase (LDH) levels, with no laboratory evidence of active hemolysis. Further metabolic evaluation revealed decreased 25-hydroxyvitamin D [25(OH)D] at 15.1 nmol/L (NR, 47.7–144 nmol/L) and markedly elevated bone turnover markers: bone-specific alkaline phosphatase (B- ALP, 38.01 μg/L; NR ≤22.40 μg/L), osteocalcin (>300 ng/mL; NR 15–46 ng/mL), and β-C-terminal telopeptide of type I collagen (β-CTX, >6.000 ng/mL; NR 0.113–0.999 ng/mL), consistent with high bone turnover. Whole-body bone scintigraphy (SPECT) demonstrated increased radiotracer uptake in bilateral long bones, axial skeleton, skull (“helmet sign”), and sternum (“tie sign”), indicative of a “super scan” pattern.

**Figure 1:**
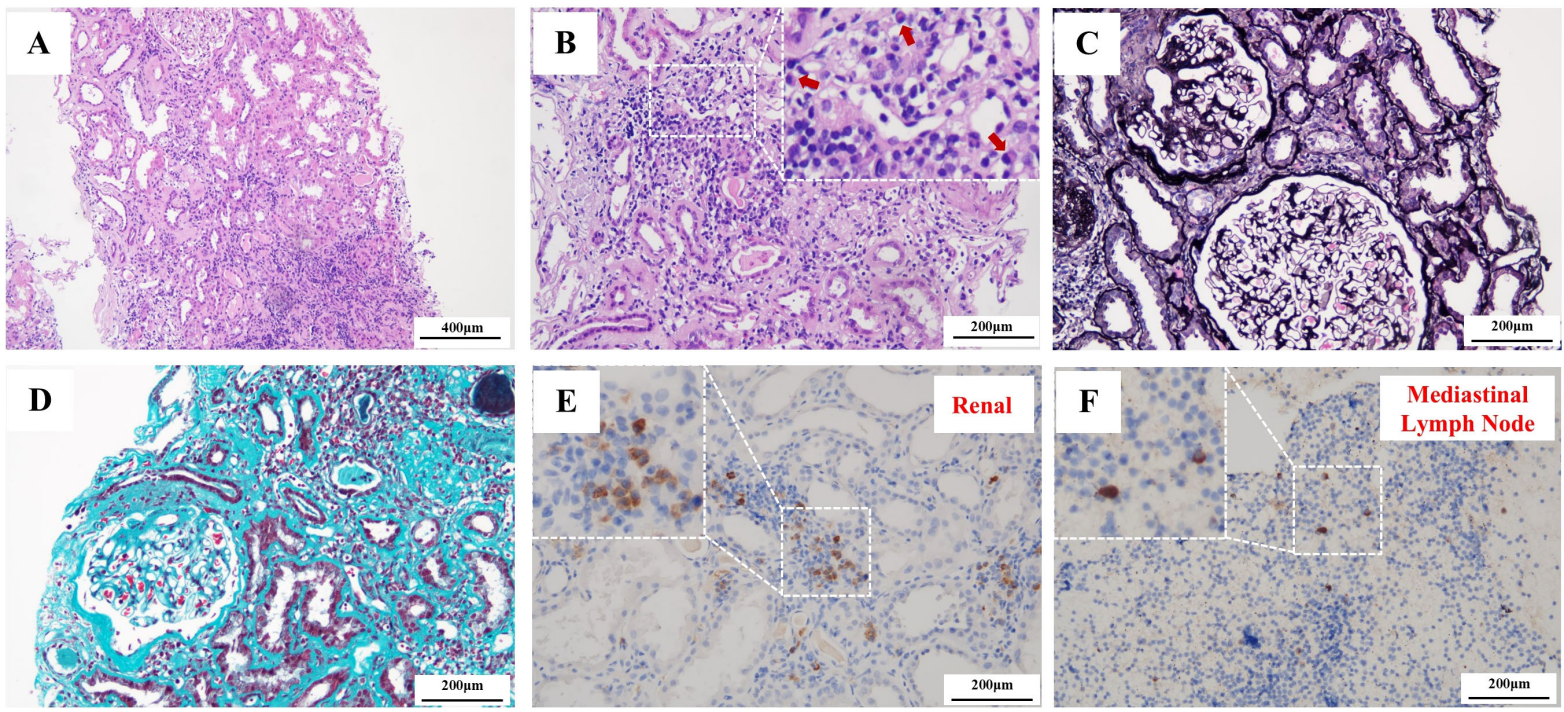
Renal and lymph nodes biopsy. **(A)** Hematoxylin–eosin (HE) staining of renal tissue shows tubular epithelial brush border loss and tubular dilation (scale bar = 400 μm); **(B)** HE staining demonstrates lymphocytic and plasma cell infiltration (red arrow indicates plasma cells) (scale bar = 200 μm); **(C)** Periodic acid–silver methenamine (PASM) staining reveals enlarged glomeruli with focal segmental sclerosis lesions (scale bar = 200 μm); **(D)** Masson’s trichrome staining highlights interstitial fibrosis (scale bar = 200 μm); **(E)** Immunohistochemistry for IgG4 in renal tissue shows >30 IgG4-positive plasma cells per HPF (scale bar = 200 μm); **(F)** Mediastinal lymph node biopsy with IgG4 staining demonstrates focal positivity, with <5 IgG4- positive plasma cells per HPF(scale bar = 200 μm).

Renal biopsy revealed the following findings. A total of approximately 15–16 glomeruli, of which 2 showed global sclerosis. Hematoxylin and eosin (HE) staining showed interstitial inflammatory cell infiltration and features of acute tubular injury, including loss of brush borders and tubular dilatation (**Figure 1A**). Lymphocytic and plasmocytic infiltration was noted, with plasma cells highlighted by red arrows (**Figure 1B**). Periodic acid–Schiff methenamine (PASM) staining demonstrated focal segmental glomerulosclerosis (**Figure 1C**), while Masson’s trichrome staining revealed interstitial fibrosis (**Figure 1D**). Immunohistochemical staining of the renal tissue revealed a maximum of approximately 30 IgG4-positive plasma cells per high-power field (HPF), with an IgG4^+^/IgG^+^ plasma cell ratio of 45% (**Figure 1E**), however, typical storiform fibrosis or obliterative phlebitis were not observed. Additionally, due to the presence of enlarged mediastinal and bilateral hilar lymph nodes, an endobronchial ultrasound (EBUS) examination was performed, which did not show evidence of malignancy. IgG4 staining of the mediastinal lymph nodes revealed only scattered positive cells, fewer than 5 per HPF, possibly due to the limited tissue sample (**Figure 1F**). PET/CT revealed inflammatory or reactive changes in the mediastinal, cervical, and abdominal lymph nodes, as well as in the bilateral submandibular glands, without evidence of malignancy. A submandibular gland biopsy was recommended but declined by the patient.

Based on the multi-organ involvement, histopathological findings, and serological results, the following diagnoses were established: a. IgG4-related disease involving the kidneys and lymph nodes. b. Hypercalcemia. c. Acute kidney injury (AKI), likely secondary to IgG4-related disease and/or hypercalcemia.

At the time of admission, hypercalcemia was initially managed with intravenous hydration, calcitonin, and loop diuretics, resulting in a gradual decrease in serum calcium. After the diagnosis was established, oral prednisone was initiated at 40 mg daily (0.8 mg/kg) for IgG4-related renal disease. The patient’s symptoms and renal function improved significantly and she was discharged on March 25. At follow-up on April 8 (day 20), sCr had decreased to 175 μmol/L and calcium to 2.85 mmol/L. By May 29, sCr was 140 μmol/L and calcium had normalized to 2.20 mmol/L with bone turnover markers declining accordingly. The patient is currently maintained on prednisone 15 mg/day. At the follow-up on September 4, 2025, sCr was 146 μmol/L, serum calcium was 2.28 mmol/L, and serum IgG4 was 3.375 g/L. Her condition continues to show favorable progression under regular monitoring. (**Supplementary Figure S2**).

## Discussion

IgG4-RD is a multisystem disorder characterized by dense lymphoplasmacytic infiltration, storiform fibrosis, obliterative phlebitis, and often elevated serum IgG4 levels (6, 7). Diagnosis of IgG4-related kidney disease requires exclusion of other causes of tubulointerstitial nephritis, including autoimmune diseases, infections, and malignancies. Clinical features may overlap with systemic lupus erythematosus, Sjögren’s syndrome, or sarcoidosis, while IgG4-positive cells can also be seen in ANCA-associated vasculitis, Castleman disease, and lymphoproliferative disorders (1, 6). In this case, the clinical, serological, imaging, and histopathological findings support the diagnosis of IgG4-RD.

IgG4-RD presenting with hypercalcemia is rarely reported. In this patient, both hypercalcemia and IgG4-RD may have contributed to the development of AKI and the relationship between hypercalcemia and IgG4-RD remains unclear, with only limited reports available. Hypercalcemia most commonly occurs in patients with primary hyperparathyroidism (PHPT, usually caused by parathyroid adenoma) or malignancy, which together account for more than 90% of cases (8), and it may also result from medication use. In this case, however, none of these potential causes were present. Existing literature classifies IgG4-RD–associated hypercalcemia into parathyroid hormone (PTH)-dependent and PTH-independent categories (9). The former may result from coincidental parathyroid adenoma in patients with IgG4-RD or from circulating IgG4 autoantibodies that inactivate calcium-sensing receptors (10). For PTH- independent cases, the underlying pathogenesis remains incompletely understood. A previous report described an IgG4-RD patient with severe hypercalcemia and normal PTH who died on day 16 of hospitalization; autopsy revealed increased osteoclasts and metastatic calcification, though an undetected malignancy could not be excluded (11). Another case involved marked elevation of serum IgG4 with polyclonal hypergammaglobulinemia and hypercalcemia, accompanied by low PTH and 25(OH)D, but without histopathological confirmation (9).

Recently, studies have highlighted that innate immune cells, particularly macrophages, may play a role in the initiation and progression of fibrosis in IgG4-RD (12–14). Several studies have shown that in IgG4-RD, activated macrophages are skewed toward differentiation into profibrotic M2 macrophage (15). Moreover, M2 macrophages derived from patients with IgG4-RD have been reported to overexpress innate immune receptors (16). Scholars have confirmed extensive infiltration of CD163-positive M2 macrophages in the salivary glands of patients with IgG4-RD, suggesting that these macrophages contribute to tissue fibrosis through the production of profibrogenic factors (17, 18).

**Figure 2:**
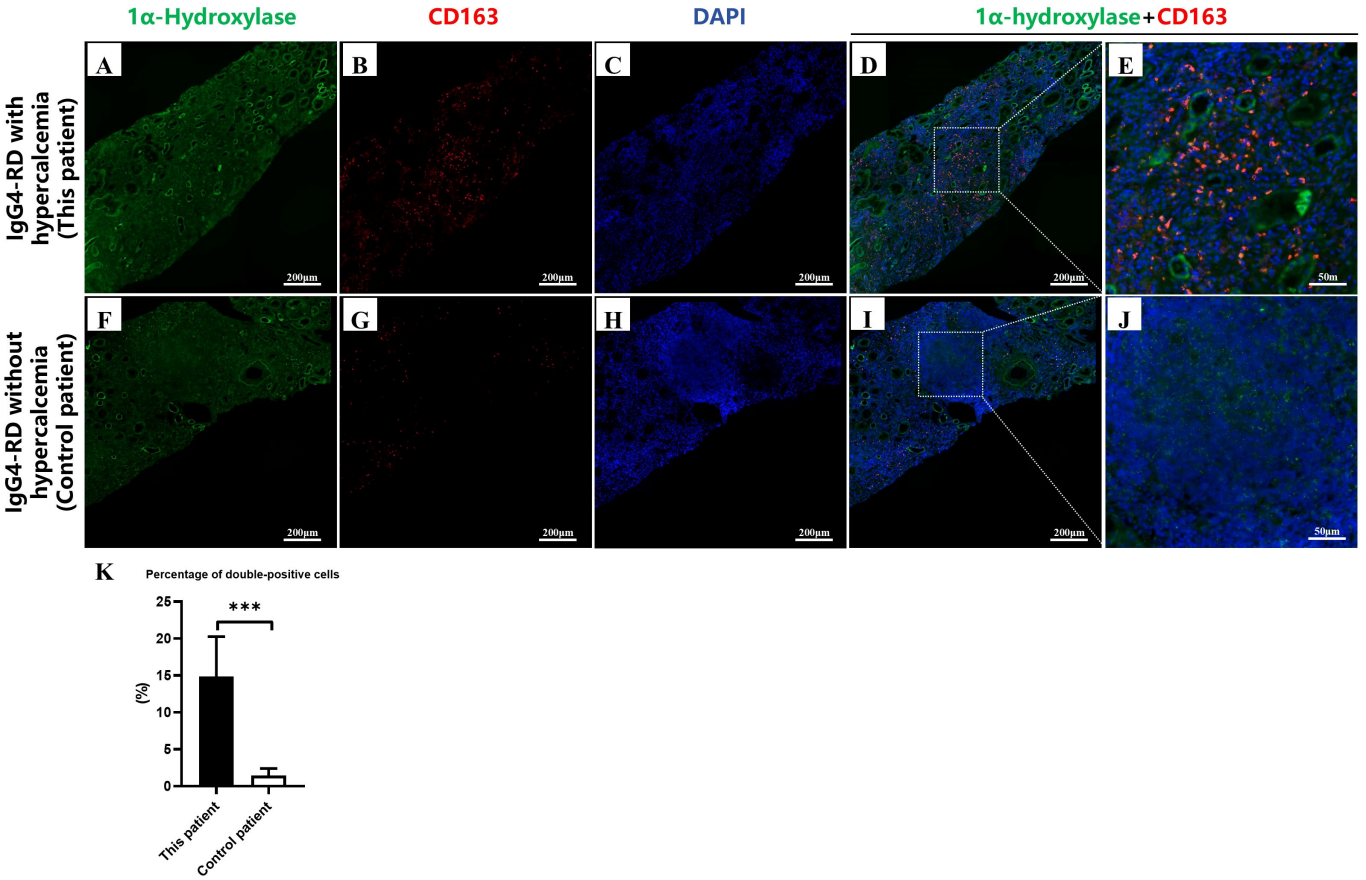
Immunofluorescence staining of renal tissue from IgG4-RD patients with and without hypercalcemia. **(A–D)** Kidney biopsy specimen from an IgG4-related disease (IgG4-RD) patient with hypercalcemia (scale bar = 200 μm). Strong expression of 1α-hydroxylase **(A)** and abundant CD163-positive macrophages **(B)** are observed. DAPI staining shows the nuclei **(C)**. Dual immunofluorescence **(D)** reveals extensive colocalization of 1α- hydroxylase and CD163 (yellow signals in the inset). **(E)** is a magnified view of the area shown in D (scale bar = 50 μm). **(F–I)** Kidney biopsy specimen from an IgG4-RD patient without hypercalcemia (scale bar = 200 μm). Compared with hypercalcemic IgG4-RD, weaker 1α-hydroxylase expression **(F)** and fewer CD163- positive macrophages **(G)** are detected. DAPI staining **(H)** and Dual immunofluorescence **(I)** show limited colocalization of 1α-hydroxylase and CD163. **(J)** is a magnified view of the area shown in I (scale bar = 50 μm). **(K)** Quantification of double-positive cells, shown as the percentage of CD163 and 1α-hydroxylase co-expressing cells (number of HPFs examined = 6).

In normal kidney tissues, macrophages generally do not exhibit significant expression of 1α-hydroxylase, with proximal tubular epithelial cells in the kidney being its primary source. In granulomatous diseases such as sarcoidosis, macrophages can overexpress 1α-hydroxylase, thereby enhancing the conversion to active 1,25(OH)_2_D, increasing intestinal calcium absorption, and ultimately leading to hypercalcemia (4, 19). In the present case, focal cellular aggregation was observed in the renal biopsy specimens. Dual immunofluorescence staining for M2 macrophages (CD163) and 1α-hydroxylase demonstrated co-localization of 1α-hydroxylase with CD163-positive cells within these areas (**Figures 2A–E**). In contrast, using renal tissue from IgG4-RD patients without hypercalcemia as controls (**Figures 2F–J**), showed no co-localization of CD163 and 1α-hydroxylase in the areas.

These findings support the hypothesis that macrophages, particularly CD163-positive cells, drive “non-classical” vitamin D activation. Similar to sarcoidosis, macrophages within IgG4-RD lesions may bypass tubular regulation and aberrantly synthesize excessive 1,25(OH)_2_D, thereby increasing intestinal calcium absorption and promoting bone calcium mobilization, ultimately resulting in hypercalcemia. This observation not only suggests a potential mechanism underlying IgG4-RD–associated hypercalcemia but also suggests that macrophages may represent a potential therapeutic target. Due to the limited amount of renal tissue available, further investigations could not be performed.

The polarization and functional properties of macrophages are highly context-dependent, exhibiting marked plasticity and disease-specific reprogramming within particular pathological microenvironments. In IgG4-RD, macrophages shift toward a pro-fibrotic “M2-like” phenotype (17, 18). These tissue-infiltrating M2 macrophages are characterized by enhanced innate immune receptor expression and production of profibrotic mediators. Meanwhile, the expression of 1α-hydroxylase—a key enzyme responsible for vitamin D activation—in macrophages is also subject to complex contextual regulation. Although *in vitro* studies indicate that M1-polarized macrophages express higher levels of 1α-hydroxylase than M2 macrophages (20), the *in vivo* microenvironment of IgG4-related tubulointerstitial nephritis is dominated by CD163^+^ M2-like macrophages, within which 1α-hydroxylase was observed to co-localize. This suggests that although the per-cell expression level of 1α-hydroxylase in M2-like macrophages may be lower than that in M1 macrophages, their numerical predominance within the renal interstitium positions them as the predominant and functionally significant source of extrarenal vitamin D activation in IgG4-RD.

Another notable finding in our patient was a positive direct Coombs test, despite the absence of polyclonal hypergammaglobulinemia or evidence of active hemolysis. The positive direct Coombs test, together with the markedly elevated rheumatoid factor, is likely a manifestation of the immune dysregulation inherent to IgG4-RD rather than a distinct co-existing autoimmune hemolytic anemia. Mild or isolated autoimmune serological abnormalities have been described in IgG4-RD and are thought to arise from dysregulated B-cell and plasmablast activation rather than organ-specific autoimmune processes (21). Thus, the Coombs positivity in this case is best interpreted as an epiphenomenon of systemic immune activation rather than an indicator of clinically meaningful hemolysis.

For the treatment of IgG4-RD, long-term management—particularly in patients with renal involvement—remains challenging. After induction of remission with prednisone, a gradual taper over several months is recommended; however, relapse is common, occurring in up to 30% of patients following dose reduction or discontinuation (22). In patients who are steroid-dependent, steroid-resistant, or experience recurrent relapses, steroid-sparing therapy is indicated. Rituximab, a B-cell–depleting monoclonal antibody, has emerged as the preferred second-line agent for both induction and maintenance of remission in IgG4-RD, particularly in those with inadequate response to glucocorticoids or intolerance to steroid therapy (23). Other immunosuppressants, such as mycophenolate mofetil and azathioprine, have been used but generally demonstrate lower efficacy. Combination therapy with glucocorticoids, conventional immunosuppressive agents, and rituximab has been associated with higher remission rates and reduced relapse risk, with rituximab maintenance providing superior prevention of disease recurrence (24).

## Conclusion

This case report describes a patient with IgG4-RD initially presenting as acute kidney injury (AKI) and hypercalcemia, thereby expanding the recognized clinical spectrum of IgG4-RD. In patients with unexplained AKI and hypercalcemia, IgG4-RD should be considered in the differential diagnosis. The association between IgG4-RD and hypercalcemia should be interpreted with caution, and the diagnosis should only be made after excluding other potential causes. Early measurement of serum IgG4 and timely tissue biopsy are crucial for prompt diagnosis and initiation of targeted therapy, which may improve clinical outcomes. Furthermore, long-term follow-up and appropriate therapeutic adjustments are essential for effective disease management.

## Data Availability

The original contributions presented in the study are included in the article/supplementary material. Further inquiries can be directed to the corresponding author.

## Supplementary material

Supplementary Figure 1 Chest CT showed bilateral patchy pulmonary infiltrates with interstitial changes, multiple enlarged mediastinal and bilateral hilar lymph nodes.

Supplementary Figure 2 Timeline of patient serum creatinine and calcium changes.
