# Supplementary figures and images for: IgG4-related disease presenting with hypercalcemia: case report and mechanistic insights
Source: Front Immunol. 2025 Dec 10;16:1720791. doi: 10.3389/fimmu.2025.1720791 (PMC12727997; doi:10.3389/fimmu.2025.1720791)

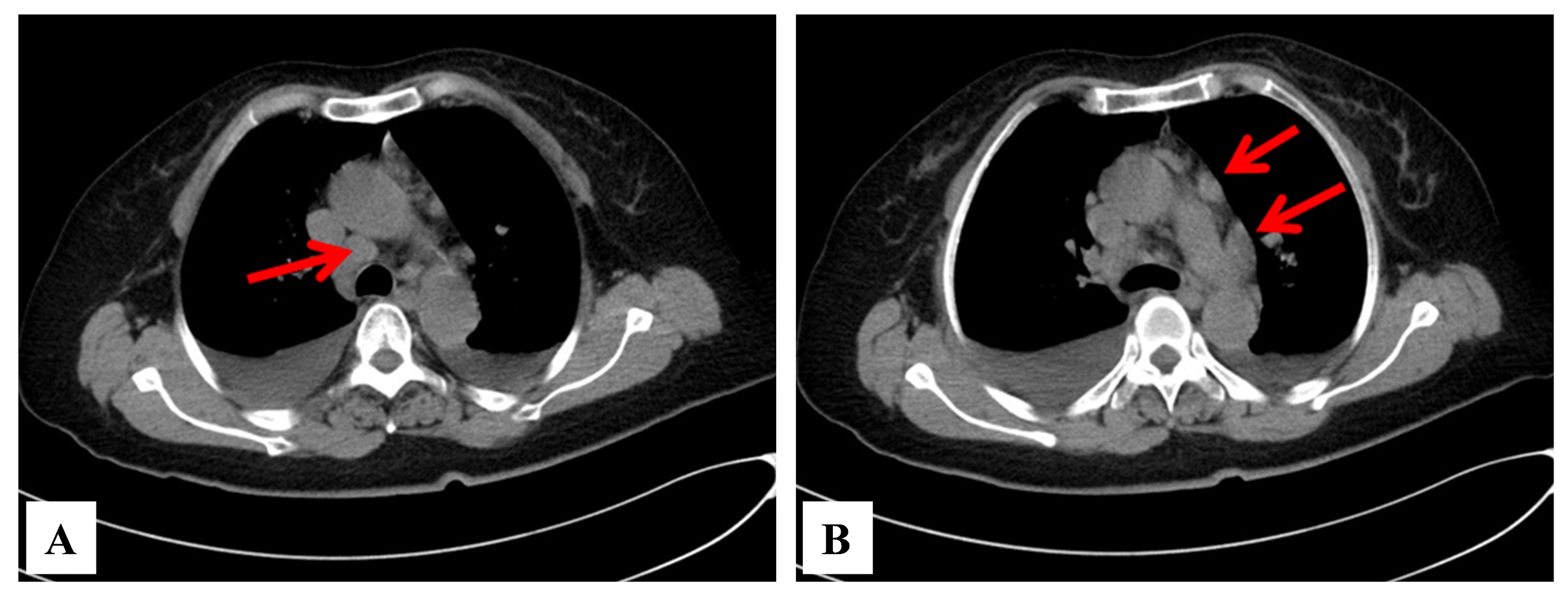

Supplement: Supplementary Figure 1 — Chest CT showed bilateral patchy pulmonary infiltrates with interstitial changes, multiple enlarged mediastinal and bilateral hilar lymph nodes. [file Image1.jpeg]

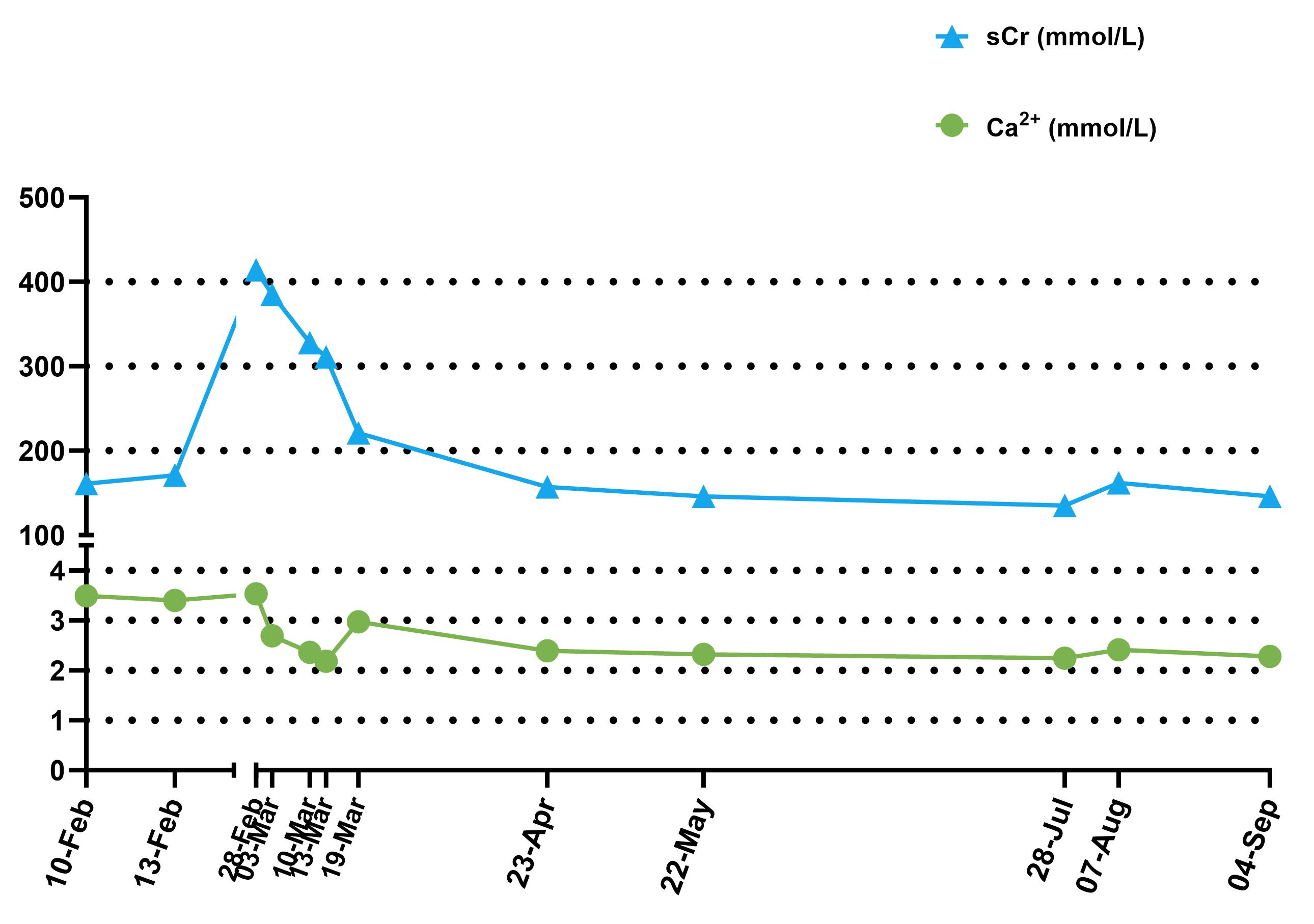

Supplement: Supplementary Figure 2 — Timeline of patient serum creatinine and calcium changes. [file Image2.jpeg]
