# Supplementary material for: IgG4-related disease presenting with hypercalcemia: case report and mechanistic insights
Source: Front Immunol. 2025 Dec 10;16:1720791. doi: 10.3389/fimmu.2025.1720791 (PMC12727997; doi:10.3389/fimmu.2025.1720791)
Supplement: Supplementary file 3 [file DataSheet1.docx]

**METHODS**

**Immunofluorescence Staining**

Paraffin-embedded renal biopsy sections (4 μm) were deparaffinized and rehydrated. Antigen retrieval was performed in EDTA buffer (pH 9.0) using a microwave at high power for 8 min twice. After blocking with goat serum for 30 minutes at 37 °C, dual immunofluorescence staining was performed. Sections were incubated overnight at 4 °C with a mixture of primary antibodies: mouse anti-human CD163 monoclonal antibody (ZSGB-BIO, ZM-0428; dilution 1:150) and rabbit recombinant monoclonal CYP27B1 (1α-hydroxylase) antibody (Abcam, ab206655; dilution 1:4000). After washing, sections were incubated for 1 hour with Alexa Fluor 647–conjugated goat anti-mouse IgG and Alexa Fluor 488–conjugated goat anti-rabbit IgG secondary antibodies (Jackson Immuno Research; dilution 1:200). Nuclei were counterstained with DAPI. Images were acquired using a Zeiss Imager Z2.
